# *n*-3 Polyunsaturated Fatty Acids Impede the TCR Mobility and the TCR–pMHC Interaction of Anti-Viral CD8^+^ T Cells

**DOI:** 10.3390/v12060639

**Published:** 2020-06-12

**Authors:** Younghyun Lim, Seyoung Kim, Sehoon Kim, Dong-In Kim, Kyung Won Kang, So-Hee Hong, Sang-Myeong Lee, Hye Ran Koh, Young-Jin Seo

**Affiliations:** 1 Department of Life Science, Chung-Ang University, Seoul 06974, Korea; acrosssv@cau.ac.kr (Y.L.); edc1213@hanmail.net (S.K.); kdi0908@cau.ac.kr (D.-I.K.); 2 Department of Chemistry, Chung-Ang University, Seoul 06974, Korea; sehoonzz@cau.ac.kr; 3 Division of Biotechnology, College of Environmental and Bioresources, Jeonbuk National University, Iksan 54596, Korea; gp1900@naver.com (K.W.K.); leesangm@jbnu.ac.kr (S.-M.L.); 4 Department of Biotechnology, the Catholic University of Korea, Bucheon 14662, Korea; hongsohee@gmail.com

**Keywords:** omega-3, CD8^+^ T cells, LCMV, TCR-pMHC interaction

## Abstract

The immune-suppressive effects of omega-3 (*n*-3) polyunsaturated fatty acids (PUFAs) on T cells have been observed via multiple in vitro and in vivo models. However, the precise mechanism that causes these effects is still undefined. In this study, we investigated whether *n*-3 PUFAs regulated T cell receptor (TCR) and peptide-major histocompatibility complex (pMHC) interactions. The expansion of anti-viral CD8^+^ T cells that endogenously synthesize *n*-3 PUFAs (FAT-1) dramatically decreased upon lymphocytic choriomeningitis virus (LCMV) infection in vivo. This decrease was not caused by the considerable reduction of TCR expression or the impaired chemotactic activity of T cells. Interestingly, a highly inclined and laminated optical sheet (HILO) microscopic analysis revealed that the TCR motility was notably reduced on the surface of the FAT-1 CD8^+^ T cells compared to the wild type (WT) CD8^+^ T cells. Importantly, the adhesion strength of the FAT-1 CD8^+^ T cells to the peptide-MHC was significantly lower than that of the WT CD8^+^T cells. Consistent with this result, treatment with docosahexaenoic acid (*DHA*), one type of *n*-3 PUFA, significantly decreased CD8^+^ T cell adhesion to the pMHC. Collectively, our results reveal a novel mechanism through which *n*-3 PUFAs decrease TCR-pMHC interactions by modulating TCR mobility on CD8^+^ T cell surfaces.

## 1. Introduction

Omega-3 polyunsaturated fatty acids (*n*-3 PUFAs) are immunological modulators that can prevent unwanted hyper-immune responses [1,2]. Their anti-inflammatory effects have been reported for various autoimmune diseases such as asthma, inflammatory bowel disease, and rheumatoid arthritis [3,4,5,6,7,8]. Furthermore, excessive or undesired immune responses caused by bacterial and viral infections can be suppressed with a *n*-3 PUFA treatment [9,10,11,12,13,14]. Several previous studies have suggested that dampening T cell responses may be an immunosuppressive mechanism of the *n*-3 PUFAs. The activation of CD4^+^ and CD8^+^ T cells was blunted after treatment with the *n*-3 PUFAs [3,13,15,16,17,18,19,20]. Furthermore, the incorporation of the *n*-3 PUFAs into the membrane induces changes in membrane composition and microdomain organization [21,22] that may result in suppressed T cell functionality. However, the precise mechanism of how *n*-3 PUFAs influence T cell responses is unidentified.

CD8^+^ T cells recognize antigens through physical contact between T cell receptors (TCRs) and the peptide-major histocompatibility complex (pMHC) on antigen-presenting cells. This triggers T cell activation and leads to the eradication of the damaged target cells (e.g., tumor and virus-infected cells). Therefore, investigating the mechanism that regulates the physical interaction between the TCR and the pMHC is important in understanding the overall T cell-mediated immune responses. Although TCR affinity cannot be altered by somatic hypermutation, membrane characteristics such as fluidity and lipid rafts can influence the degree of bond formation between the TCR and the pMHC [23,24,25]. Since *n*-3 PUFAs are known to affect membrane characteristics [21,22], the TCR-pMHC interaction may be regulated by *n*-3 PUFAs, which may subsequently influence CD8^+^ T cell functionality.

The lymphocytic choriomeningitis virus (LCMV) is a member of the *Arenaviridae* family of viruses and has a negative-strand RNA genome [26]. An acute LCMV (strain Armstrong) infection in mice strongly triggers the activation of anti-viral CD8^+^T cells, leading to a rapid viral clearance within seven to eight days post-infection [27,28]. Therefore, the infection of laboratory mice with the LCMV is a useful animal model to investigate the underlying mechanisms of the anti-viral CD8^+^ T cell response.

In this study, we investigated the potential role of *n*-3 PUFAs in regulating the TCR-pMHC interactions. Remarkably, we found that *n*-3 PUFAs reduced TCR mobility on the surface of CD8^+^ T cells, which potentially causes decreased TCR-pMHC adhesion. 

## 2. Materials and Methods

### 2.1. Mice

C57BL/6 mice (DBL Korea, Yongin, Korea) (CD45.1-/CD45.2+), LCMV glycoprotein (GP) 33-41-specific T cell receptor (TCR) transgenic (P14) mice (donated by Dr. Sang-Jun Ha, Yonsei University, Seoul, Korea) (CD45.1+/CD45.2-), *n*-3 PUFA desaturase knock-in (FAT-1) mice (kindly provided by Dr. JX Kang of Harvard Medical School, Boston, USA), and FAT-1 P14 mice (CD45.1+/CD45.2+) were used. FAT-1 transgenic mice are capable of producing n-3 PUFAs since these mice express the *fat-1* gene that encodes the n-3 PUFA desaturase (from *C. elegans*) to convert n-6 into n-3 fatty acids [29]. The expression of the *fat-1* gene was screened by *fat-1* targeted PCR. The genetic background of all the mice used in this study was C57BL/6. All the mice were maintained and bred in individual, ventilated, and closed cage systems. Experiments requiring in vivo LCMV infection were performed in an animal biosafety level 2 (ABLS2) facility (Korea Zoonosis Research Institute, KoZRI, Iksan, Korea). All experimental protocols were approved by the Institutional Animal Care and Use Committee at Chonbuk National University (CMNU 2019-013).

### 2.2. Virus and Infection

LCMV Clone 13 (Cl 13) and Armstrong (Arm) were amplified in baby hamster kidney cells (BHK) (American Type Culture Collection, Manassas, VA, USA) [30]. For the in vivo experiment, mice were infected with 2 × 10^5^ focus forming units (FFUs) of LCMV Arm or 1.5 × 10^6^ FFU of LCMV Cl 13.

### 2.3. Reagents and Antibodies

Mouse splenocytes were cultured in a complete RPMI-1640 medium (GenDEPOT, Katy, TX, USA) supplemented with fetal bovine serum (10%, Hyclone, South Logan, UT, USA) and 1% penicillin/streptomycin (Welgene, Gyeongsan, Korea). Anti-mouse TCR-β-PE, CD45.1-Percp Cy5.5, CD3-APC Cy7, CD8-FITC, CD44-PE, and IFN-γ-FITC antibodies as well as CFSE cell proliferation tracing dye were purchased from Tonbo Bioscience (San Diego, CA, USA). Anti-mouse TNF-α-PE Cy7, APC-conjugated streptavidin, and PE-conjugated streptavidin were purchased from Biolegend (San Diego, CA, USA). Gp^33-41^ class I pMHC tetramer was provided by the NIH Tetramer Core Facility (Atlanta, GA, USA).

### 2.4. Isolation of CD8^+^ Cells

CD8^+^ cells were purified using a MojoSort mouse CD8^+^ T cell isolation kit (Biolegend, San Diego, CA, USA) according to the manufacturer’s instructions. Briefly, the splenocytes were incubated with a CD8^+^ negative selection antibody cocktail and incubated with streptavidin-coated metal beads. The desired cells were purified with a magnet, and the unwanted cells were washed away. CD8^+^ T cell purity (>95%) was confirmed via flow cytometry. 

### 2.5. In Vitro Activation of CD8^+^ Cells

The splenocytes were incubated in the presence of GP^33-41^ peptide (1 μg/mL) and 6 μg/mL of LPS (Sigma-Aldrich, Saint Louis, MO, USA) for six days. Two days after the initial stimulation, 12.5 U/mL of murine IL-2 (Peprotech, Rocky Hill, NJ, USA) was added to the media. The CD8^+^ cells were isolated with a MojoSort mouse CD8^+^ T cell isolation kit (Biolegend, San Diego, CA, USA) before use.

### 2.6. Generation of Bone Marrow-Derived Dendritic Cells

The bone marrow cells obtained from the femur of naïve C57BL/6 mice were transferred to a 100 mm petri dish and cultured in an RPMI medium supplemented with 200 U/mL of mGM-CSF (Peprotech, Rocky Hill, NJ, USA). Six days later, the cells were analyzed for the expression of CD11b, CD11c, and MHC II by flow cytometry before further experiments.

### 2.7. Trans-Well Chemotaxis Assay

Purified CD8^+^ T cells were resuspended in RPMI media (2.0 × 10^6^ cells/mL), and 100 µL was added into a SPL Insert™ Hanging well (pore size: 3 um) (SPL, Pocheon, Korea). 300 µL of RPMI media with or without CCL19 (Peprotech, Rocky Hill, NJ, USA) was placed in the bottom chamber. Transferred cell numbers were normalized to the relative cell numbers.

### 2.8. pMHC-TCR Binding Assay

For in situ assessment of T cell receptor–pMHC affinity, a polystyrene 96 well plate (Nunc MaxiSorp™ flat-bottom, Invitrogen, Waltham, CA, USA) was coated with streptavidin (Sigma-Aldrich, Saint Louis, MO, USA). Multiple concentrations of gp^33-41^ class I pMHC were then added. Lastly, CD8^+^ T cells (2.0 × 10^5^ cells) were added into each well. After one hour of incubation, the plates were washed with pre-warmed RPMI media to wash out any unbound cells. The number of attached cells was counted under a light microscope. 

### 2.9. Highly Inclined and Laminated Optical Sheet (HILO) Microscopic Analysis

We diluted the CD8^+^ T cells that were stained with a PE-conjugated anti-TCR-β antibody in the imaging buffer (4 mM Trolox, 0.8% (w/v) glucose, 50 mM NaCl, 165 U/mL glucose Oxidase, 2170 U/mL catalase) for enhancing the stability of the PE during the imaging acquisition process and added the cells in the imaging chamber for monitoring TCRs of the cells using a highly inclined and laminated optical sheet (HILO) microscope [31]. The homebuilt objective total internal reflection microscope was modified to excite the cells at a highly inclined angle. The 532 nm laser (Cobolt, Sweden) excited PE-conjugated TCRs in the cells through a 60× water immersion objective (Olympus, Japan) that gathered the fluorescence emission of PE to the EMCCD camera (Andor iXon897, Andor Technology, Belfast, UK). Then, the recorded fluorescence movies that were obtained with the EMCCD camera were analyzed using the ImageJ (https://imagej.nih.gov/ij/) software (NIH, Bethesda, MD, USA).

### 2.10. Statistical Analyses

All statistical significances were calculated using Student’s t-test. The error bars indicate the SEM (standard error of the mean). The calculated mean values were compared and defined as statically significant or not. All the experiments were repeated independently at least three times.

## 3. Results

### 3.1. n-3 PUFAs Reduce in Vivo Expansion of Anti-Viral CD8^+^ T Cells

**Figure 1:**
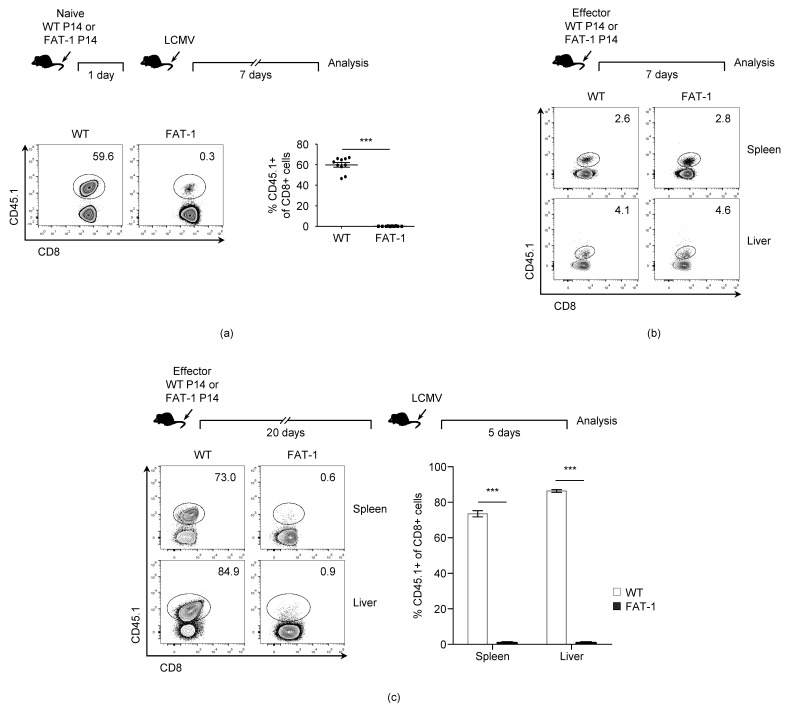
The *n*-3 polyunsaturated fatty acid (PUFA) decreases the CD8 response against the lymphocytic choriomeningitis virus (LCMV) infection. (**a**) 1.0 × 10^5^ wild type (WT) P14 or FAT-1 P14 cells were transferred into the naïve WT mice via tail vein intravenous injection followed by an intraperitoneal infection with 2.0 × 10^5^ focus forming units (FFUs) of the LCMV Armstrong after 24 h. Seven days after infection, the mice were sacrificed and splenocytes were analyzed. (**b**) 1.5 × 10^6^ in vitro-activated (effector) WT P14 or FAT-1 P14 cells were transferred intravenously into the naïve WT mice. Seven days after the transfer, the mice were sacrificed to analyze the P14 T cells in the livers and spleens. (**c**) 1.5 × 10^6^ in vitro-activated WT P14or FAT-1 P14 cells were transferred intravenously into the naïve WT mice. Twenty days after transfer, the mice were challenged with 1.5 × 10^5^ FFUs of LCMV clone 13 via intravenous injection. Five days after challenge, the mice were sacrificed to analyze P14 T cells in the livers and spleens. ***, *p* < 0.001.

In agreement with a previous report [13], the P14 T cells endogenously synthesizing the *n*-3 PUFAs (FAT-1 P14) that were adoptively transferred into the naïve wild type (WT) mice displayed dramatically reduced expansion capability upon LCMV infection in vivo when compared to WT P14 cells (Figure 1a). Since the *n*-3 PUFAs are involved in the survival of diverse cell types including tumor cells [32,33,34], we compared the survival rates between the WT P14 and the FAT-1 P14 cells in vivo. The naïve WT P14 and FAT-1 P14 cells were stimulated with the LCMV glycoprotein 33-41 (gp33) peptide in vitro to generate effector cells that were transferred into the naïve WT mice respectively. Seven days later, the numbers of adoptively transferred WT P14 and FAT-1 P14 cells were measured in spleens. However, there was no significant reduction in the number of adoptively transferred FAT-1 P14 cells when compared to WT P14 cells. This indicates that the endogenous *n*-3 PUFAs did not reduce the survival of the effector CD8^+^ T cells (Figure 1b). Next, we investigated whether the *n*-3 PUFAs affected the memory CD8^+^ T cell response against the LCMV infection. The in vitro-generated effector WT P14 or FAT-1 P14 cells were transferred into the naïve WT mice respectively, and 20 days later, the mice were challenged with the LCMV to measure the expansion of the WT P14 and FAT-1 P14 cells, respectively. The expansion of the FAT-1 P14 cells was greatly reduced compared to WT P14 cells in both the spleen and liver, indicating that the memory anti-viral CD8^+^ T cell response was reduced by the endogenous expression of *n*-3 PUFAs (Figure 1c). Collectively, these results suggest that endogenous *n*-3 PUFAs down-regulate the expansion of CD8^+^ T cells during an LCMV infection in vivo.

### 3.2. n-3 PUFAs Do Not Affect the Intrinsic Activation and Migration Potential of CD8^+^ T Cells

**Figure 2:**
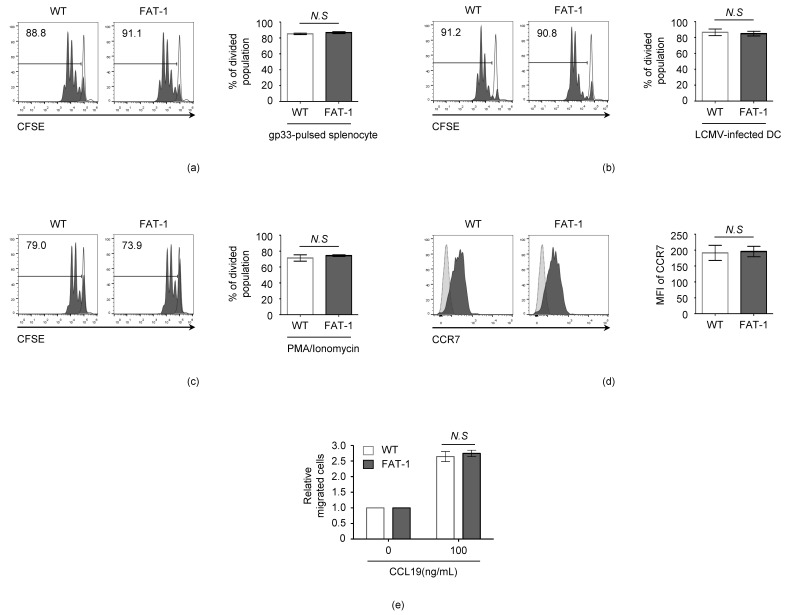
The endogenous *n*-3 PUFA does not reduce the in vitro proliferation or migration of CD8^+^ T cells. (**a**) The splenocytes from WT C57BL/6 mice were pulsed with 1 μg/mL of gp33 peptide for one hour. The pulsed cells were co-cultured with CFSE-stained WT P14 or FAT-1 P14 CD8^+^ T cells for two to three days and analyzed with flow cytometry. (**b**) The bone marrow-derived dendritic cells were infected with the LCMV Armstrong (MOI = 1). Twenty-four hours after infection, the infected dendritic cells were co-cultured with CFSE-stained WT P14 or FAT-1 P14 CD8^+^ T cells for two to three days and analyzed with flow cytometry. (**c**) The CFSE-stained WT P14 or FAT-1 P14 CD8^+^ T cells were stimulated with PMA (20 ng/mL) and ionomycin (500 ng/mL) for two to three days and analyzed with flow cytometry. (**d**) The Naïve WT P14 or FAT-1 P14 CD8^+^ T cells were stained with a CCR7 chemokine receptor and analyzed with flow cytometry. The light-gray histogram plot represents the fluorescent minus one (FMO) control. (**e**) The Naïve WT P14 or FAT-1 P14 CD8^+^ T cells were resuspended in 0.1% BSA RPMI medium to a concentration of 2.0 × 10^5^ cells/100 μL. Next, 100 μL of cells were loaded into the 3 μm pore upper well. The lower well was filled with 500 μL of complete RPMI medium enriched with 100 ng/mL of CCL19. Twenty hours later, the number of cells in the lower well was counted to calculate the relative migrated cells. *N.S*, not significant.

Next, we examined whether decreased in vivo FAT-1 P14 proliferation was reproducible in in vitro peptide stimulation or infection conditions. The proliferation of the WT P14 and FAT-1 P14 cells was measured by the loss of CFSE fluorescence under the stimulation of the LCMV gp33 peptide (Figure 2a) or LCMV-infected dendritic cells (DC) (Figure 2b). Interestingly, the proliferation rates of the WT P14 and FAT-1 P14 cells were not significantly different when the cells were stimulated with either gp33 peptide (Figure 2a) or LCMV-infected DC (Figure 2b).

*n*-3 PUFAs may influence the intrinsic activation potential of CD8^+^ T cells, resulting in a differential expansion pattern between WT P14 and FAT-1 P14 CD8^+^ T cells. To test this hypothesis, the WT P14 and FAT-1 P14 CD8^+^ T cells were treated with PMA/ionomycin that diffuses directly into the cytoplasm to activate the Protein Kinase C and NFAT signaling pathways [35]. However, no significant differences between the WT P14 and FAT-1 P14 CD8^+^ T cell proliferation (CFSE+ cells, WT: 71% ± 4%; FAT-1: 74% ± 1%) were observed (Figure 2c). The chemotactic migration of naïve CD8^+^ T cells into secondary lymphoid organs is a critical step in initiating an anti-viral CD8^+^ T cell response. Therefore, we tested whether *n*-3 PUFAs influence the chemotactic migration ability of CD8^+^ T cells. The expression level of a major lymph node trafficking receptor, CCR7, was not significantly different between the WT and FAT-1 CD8^+^ T cells (Figure 2d). Furthermore, when the T cell migration ability was measured with the trans-well assay, the chemotactic migration in response to CCL19 (a CCR7 ligand) was comparable between the WT and FAT-1 CD8^+^ T cells (Figure 2e). These results indicate that endogenous *n*-3 PUFAs do not affect the intrinsic activation and migration potential of anti-viral CD8^+^ T cells.

### 3.3. n-3 PUFAs Decrease TCR Mobility on CD8^+^ T Cell Membrane

**Figure 3:**
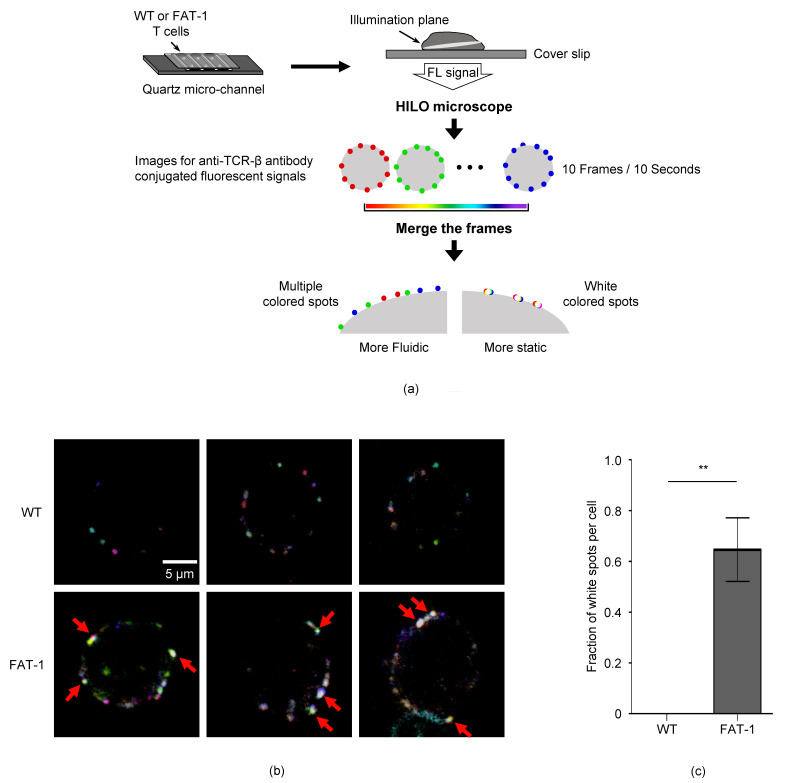
The endogenous *n*-3 PUFA decreases T cell receptor (TCR) mobility on CD8^+^ T cells. (**a**) An experimental scheme is shown. The WT or FAT-1 CD8^+^ T cells were stained with an anti-mouse TCR-β-PE antibody and loaded into a quartz micro-channel, followed by analysis using a highly inclined and laminated optical sheet (HILO) microscope. (**b**) Three representative merged images for the TCRs on the WT or FAT-1 CD8^+^ T cells are shown. The red arrow indicates the white spot. (**c**) The fraction of less mobile spots that appear as white spots in the merged image over 10 frames was calculated by counting the number of white spots and dividing it by the average number of total spots per frame. **, *p* < 0.01.

*n*-3 PUFAs are known to regulate cell membrane fluidity [36], which could affect receptor–ligand interaction [37,38]. Since TCR-pMHC interaction is prerequisite for the activation of T cells, we investigated whether endogenous *n*-3 PUFAs affect the TCR mobility of CD8^+^ T cells. To this end, the WT or FAT-1 CD8^+^ T cells that were stained with the PE-conjugated anti-TCR-β antibody were analyzed by a highly inclined and laminated optical sheet (HILO) microscope (Figure 3a). To visualize TCRs on cells, we used a home-built fluorescence microscope with HILO illumination because it provided an improved signal-to-noise ratio compared to a conventional wide-field microscope. The fluorescent movies of the WT and FAT-1 CD8^+^ T cells displayed the mobile or static fluorescent spots of the TCRs. Then, we analyzed the fluorescent movies by using a temporal color coding for the first 10 frames of each movie, where each frame has its own unique color. With the temporal color coding, the static spots tended to exhibit white colors in the merged image for the first 10 frames because the fixed spots that located at the same position over multiple time frames displayed the overlapped white colors. However, the mobile spots exhibited the unique color of each frame because it changed its position over time (Figure 3a). The whiter spots from the FAT-1 CD8^+^ T cells compared with WT cells demonstrated that the TCRs of WT cells possessed a significantly higher fluidity than the ones of the FAT-1 cells (Figure 3b,c). Thus, these results show that *n*-3 PUFAs reduced TCR mobility on the CD8^+^ T cell surface. 

### 3.4. Endogenous n-3 PUFAs Interfere with TCR-pMHC Interactions

**Figure 4:**
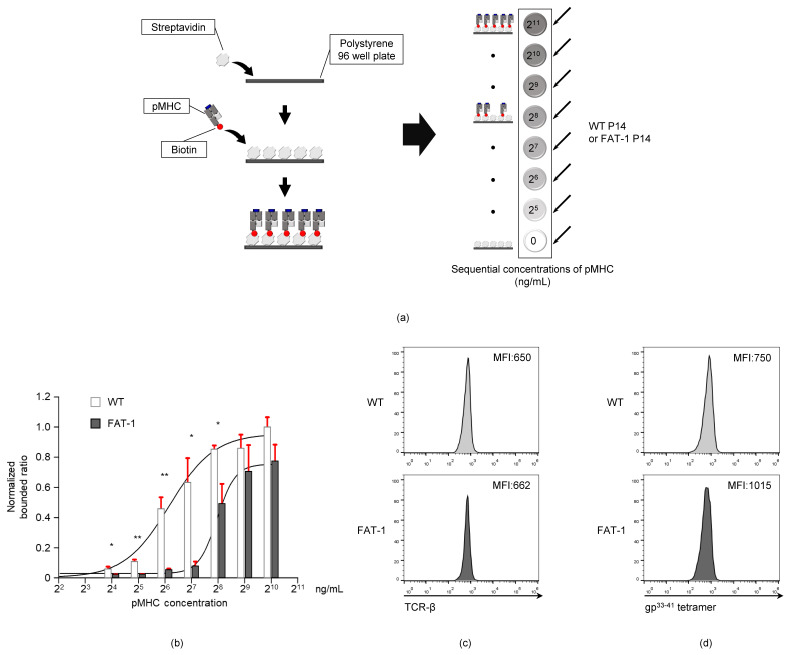
Endogenous *n*-3 PUFAs decrease the TCR-peptide-major histocompatibility complex (pMHC) interaction of anti-viral CD8^+^ T cells. (**a**) An experimental scheme is shown. A 96-well polystyrene plate was coated with 100 μL of streptavidin (10 μg/mL) and 100 μL of pMHC (1000 ng/mL to 16 ng/mL concentration range), sequentially. The WT P14 or FAT-1 P14 CD8^+^ T cells (1.0 × 10^5^ cells) were loaded onto the coated wells and incubated at 37 °C for one hour. After incubation, each well was washed with a pre-warmed RPMI medium and the attached cells were counted. (**b**) The attached WT P14 or FAT-1 P14 cells were counted and normalized against the WT P14 cells (pMHC 1000 ng/mL condition). (**c**,**d**) The mean fluorescence intensities (MFIs) for TCR expression (**c**) and gp^33-41^ tetramer staining (**d**) on WT P14 or FAT-1 P14 cells are shown. *, *p* < 0.05; **, *p* < 0.01.

Since TCR-pMHC bond formation is regulated by plasma membrane fluidity and dynamics [23,39,40], the reduced TCR mobility on the surface of CD8^+^ T cells (Figure 3) might interfere with TCR-pMHC interactions. To test this hypothesis, we developed a plate-based TCR-pMHC binding assay technique (Figure 4a). The WT P14 or FAT-1 P14 cells were added to a 96-well plate that was previously coated with various concentrations of the LCMV gp^33-41^MHC (0, 15.625, 31.25, 62.5, 125, 250, 500, and 1000 ng/mL). As the concentration of the gp^33-41^MHC complexes increased, the gp^33-41^MHC-coated bottom-attached WT P14 cell numbers increased. This was due to the antigen-specific TCR-MHC interactions (Figure 4b). Remarkably, the number of gp^33-41^MHC-binding FAT-1 P14 cells was significantly less than that of the WT P14 cells at gp^33-41^MHC concentrations of 15.625, 31.25, 62.5, 125, and 250 ng/mL (Figure 4b). Differential TCR expression between WT P14 and FAT-1 P14 cells may affect this result; therefore, we compared the TCR expression levels between the two cell types. As shown in Figure 4c, the WT P14 and FAT-1 P14 cell TCR expression levels were comparable. Similarly, the mean fluorescence intensities (MFIs) for the gp^33-41^ tetramer staining of the FAT-1 P14 cells were not higher than those of the WT P14 cells (Figure 4d). Collectively, the FAT-1 P14 cells adhered less to the plate-bound gp^33-41^MHC than the WT P14 cells, and this was not caused by differential TCR expression on the P14 cells. 

### 3.5. Docosahexaenoic Acid (DHA) Treatment Reduces CD8^+^ T Cell TCR-pMHC Bond Formation

**Figure 5:**
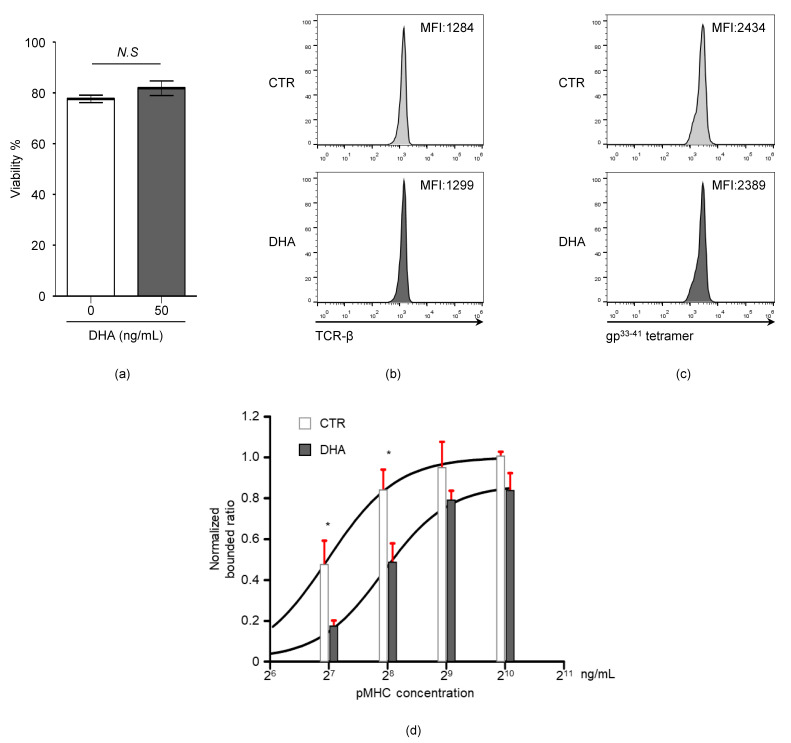
The extrinsic treatment with *n*-3 PUFA docosahexaenoic acid (DHA) decreases TCR-pMHC affinity. The WT P14 cells were untreated (control, CTR) or treated with 50 μM of DHA in the presence of 200 U/mL of IL-2 for 24 h. (a-c) Cell viability (% propidium iodide-negative cells) (**a**), the TCR expression (**b**), and gp^33-41^ tetramer staining (**c**) were analyzed by flow cytometry. (**d**) The CTR or DHA-treated cells (2.0 × 10^5^ cells) were loaded into pMHC-coated cells. After one hour of incubation, the attached cells were counted and normalized against the CTR P14 T cells (pMHC 1000 ng/mL condition). *N.S*, not significant; *, *p* < 0.05.

Our results showing that the endogenous expression of *n*-3 PUFAs decreased TCR-pMHC bond formation (Figure 4) led us to test whether the exogenous treatment of *n*-3 PUFAs is also capable of influencing TCR-pMHC interactions. To this end, the WT P14 cells were either untreated or treated with docosahexaenoic acid (*DHA*), an *n*-3 PUFA, to measure the adhesion strength between the P14 cells and the gp^33-41^MHC. The DHA treatment did not significantly reduce the viability (untreated: 77.7 ± 1.2% versus DHA: 81.8 ± 2.9%) (Figure 5a), the expression level of TCR (MFI, untreated: 1284 versus DHA: 1299) (Figure 5b), or the MFI for gp^33-41^ tetramer staining (MFI, untreated: 2434 versus DHA: 2389) (Figure 5c). However, when cells were analyzed with the plate-based TCR-pMHC binding assay, the DHA-treated P14 cells were remarkably less adhesive to the gp^33-41^MHC (Figure 5b) than the untreated P14 cells (Figure 5d). These data indicate that exogenous *n*-3 PUFA treatment also interferes with the degree of bond formation between CD8^+^ T cell TCR and pMHC. 

## 4. Discussion

Omega-3 has therapeutic potential against several immune disorders. Although its anti-inflammatory activity on CD4^+^ and CD8^+^ T cells is known to play a central role, the detailed underlying mechanism of its therapeutic potential is still unknown. The aim of this study was to identify how *n*-3 PUFAs reduced the activation of T cell responses. The physical interaction between a TCR and the cognate antigen, in the context of the MHC molecule on the target cell, leads to the activation of the T cell. Surprisingly, the TCR mobility of *n*-3 PUFA-sufficient FAT-1 CD8^+^ T cells was significantly lower than that of the WT cells. Additionally, the *n*-3 PUFAs reduced the TCR-pMHC bond formation. These results provide novel insight into how *n*-3 PUFAs regulate T cell activation by interfering with TCR-pMHC interactions.

Incorporation of *n*-3 PUFAs into the membrane alters the phospholipid composition, lipid raft formation, and cholesterol deposition in the membrane, which can change certain membrane characteristics such as fluidity [41,42]. Indeed, our results indicate that endogenous *n*-3 PUFAs reduce CD8^+^ T cell TCR mobility. Since TCR mobility is important for supplying TCRs to the immunological synapse during T cell and target cell interactions [43], TCR-pMHC interactions could be affected by the degree of TCR mobility. Our results demonstrated that TCR-pMHC interactions were significantly reduced in *n*-3 PUFA-sufficient CD8^+^ T cells. Therefore, these results support that an *n*-3 PUFA-mediated reduction in TCR mobility possibly interferes with CD8^+^ T cell TCR and antigen interactions in the context of MHC molecules on target cells. Although further investigations are required, *n*-3 PUFAs might also induce other modifications such as changes in cytoskeleton alignment, the motion of other large membrane proteins, and signaling/clustering associated with T cell adhesion, which could influence TCR-pMHC interaction.

Since *n*-3 PUFAs can affect membrane fluidity [44,45], the mobility of other proteins on the cell surface may also be changed. This might influence the interactions between other surface proteins. For example, the activation of CD4^+^ T cells also requires physical interaction between TCRs and MHC class II molecules. Therefore, the CD4^+^ T cell response might be influenced by membrane-incorporated *n*-3 PUFAs. Indeed, *n*-3 PUFAs are known to down-regulate the antigen-dependent activation of CD4^+^ T cells [46,47]. Therefore, *n*-3 PUFAs could be used to suppress the unwanted hyperactivation of both CD8^+^ and CD4^+^ T cells during infections and autoimmune diseases.

In this study, we developed a novel plate-based TCR-pMHC binding assay to confirm whether *n*-3 PUFAs regulated TCR-pMHC interactions. While the gp^33-41^ tetramer staining did not discriminate the TCR-pMHC interactions between the WT P14 and FAT-1 P14 cells, this assay result indicated that the TCR-pMHC interactions of the FAT-1 P14 cells were significantly lower than that of the WT P14 cells. Therefore, this simple assay system is a useful tool for studying the regulation of physical interactions between TCR-pMHC. Furthermore, this assay system could be used to study the interaction between other diverse surface proteins. 

Collectively, our results provide novel insight into how antigen recognition by anti-viral CD8^+^ T cells is regulated along with its clinical application.
